# Perceived clinical competence and its related factors among registered nurses employed at selected outpatient clinics in Egypt

**DOI:** 10.1371/journal.pone.0314206

**Published:** 2024-12-02

**Authors:** Rasha A. Mohamed, Mohammed Fayez J. Alharbi, Rahmah Aloufi, Nisha Abraham, Nagwa Nabeh Taref, Eman Samy Bauomy, Safa H. Alkalash, Marzouk M. Marzouk, Abeer A. Almowafy

**Affiliations:** 1 Department of Nursing, College of Applied Medical Sciences, University of Bisha, Bisha, Saudi Arabia; 2 College of Nursing, Taif University, Taif, Kingdom of Saudi Arabia; 3 Department of Community Health Nursing, Faculty of Nursing, Mansoura University, Mansoura, Egypt; 4 Department of Community Medicine and Healthcare, Faculty of Medicine, Umm Al-Qura University, Al-Qunfudha, Kingdom of Saudi Arabia; 5 Department of Family Medicine, Faculty of Medicine, Menoufia University, Menoufia, Egypt; 6 Department of Public Health and Community Medicine, Damietta Faculty of Medicine, Al-Azhar University, Cairo, Egypt; 7 International Islamic Center for Population Studies and Research, Al-Azhar University, Cairo, Egypt; Prince Sattam bin Abdulaziz University, SAUDI ARABIA

## Abstract

**Introduction:**

Nurses’ clinical competence is a significant concern in all healthcare settings due to the necessity of delivering high-quality patient care. Understanding and addressing the factors related to competence are crucial for promoting nurses’ clinical competence and ultimately improving patient outcomes. Producing and maintaining a skilled nursing workforce is essential to protect communities.

**Aim:**

This study aimed to assess the level of self-evaluated clinical competence and its correlation with demographic and occupational variables among registered nurses employed at selected outpatient clinics in Egypt.

**Materials and methods:**

The study utilized a descriptive cross-sectional design with a self-administered, two-part questionnaire that assessed participants’ demographic and occupational variables as well as perceived clinical competence in various healthcare settings. It took place at outpatient clinics of two governmental hospitals and five primary healthcare centers in Mansoura City, Egypt between January, and June 2023. A purposive sample of 450 nurses took part in this study.

**Results:**

The average score of nurses’ clinical competence was 155.3±7.2 out of 230, indicating a “moderate level”. In terms of professional behaviors and general performance, the average score for clinical competence was 48.4±3.6 and 40.7±4.1 respectively. Additionally, the average score for clinical competence regarding core and advanced nursing skills were 43.4±3.0 and 22.8±1.5 respectively. Among the domains of clinical competence, the highest average score was associated with “professional behaviors” as it forms the backbone of nursing practice. There was a highly significant relationship between the average score of clinical competence and the participant’s age, sex, level of education, and years of clinical work experience (*P*<0.001).

**Conclusion:**

Nurses perceived their level of clinical competence as moderate. To enhance nurses’ clinical competence, future studies and interventions should focus on promoting supportive work environments, providing ongoing education and training in advanced nursing skills, and the fostering development of critical thinking skills in nurses.

**Recommendations:**

Healthcare organizations should implement educational interventions to enhance nurses’ clinical competence. These interventions should include continuous professional development opportunities, mentorship programs, inclusive training initiatives, and structured feedback mechanisms. These measures will help nurses stay up-to-date with the latest practices and technologies, create a supportive learning atmosphere, and address the unique needs and challenges faced by nurses of different genders and specialties.

## Introduction

Current views on professionalism emphasize that improving healthcare quality is both a moral and professional duty for all medical practitioners, particularly nurses [1]. Professional affiliations, organizational commitment, and job attitudes are influenced by professional competence and skills [2]. Nurses’ clinical competency is a major concern in all healthcare settings because of the requirement that nurses provide high-quality patient care and the strong correlation between nurses’ competency and the success of health organizations [3]. Research findings indicate that clinical competence is positively correlated with professional confidence, quality of working life, self-efficacy, and the efficient use of clinical abilities [4] and negatively correlated with job burnout [5]. Furthermore, patient safety and satisfaction are directly associated with clinical competence [6].

To meet population demands and safeguard the public, it is imperative to produce competent nursing staff [7]. Nurses are expected to provide qualified and safe care [8]. The need to provide safe and affordable services, raise community awareness of health issues, utilize competent staff by healthcare organizations, and adapt to rapid changes in health surveillance systems are just a few of the factors contributing to the increased focus on nurses’ clinical competence [9, 10]. Furthermore, healthcare settings have a responsibility to ensure that the nurses they employ possess the necessary skills to fulfill their professional obligations and to develop appropriate improvement plans [11–13].

Clinical competence refers to the attitudes, knowledge, and skills that a newly graduated nurse must possess to perform duties related to patient care appropriately and help maintain, restore, and advance patients’ health [14]. These skills are interrelated and relevant across various nursing practice environments [15, 16]. Competencies are divided into professional and clinical categories. Professional competency includes critical elements, such as compliance with ethical standards, a thorough knowledge of healthcare legislation, strong decision-making skills, expertise in professional development, and the ability to work collaboratively [17]. Clinical competency involves the fundamental principles of nursing care, adherence to clinical guidelines, and the implementation of effective nursing interventions [18]. It also encompasses the use of technical and communication skills, knowledge, clinical reasoning, emotions, and values within a clinical setting [19, 20].

Like other nations, Egypt has a long history of preserving and advancing the health of its residents as well as providing medical care for the sick. In Egypt, nurses are in high demand and serve as the backbone of both basic healthcare and hospital services. However, there is a persistent nursing shortage in Egypt. In 2020, Egypt had 201,623 registered nursing professionals in the governmental healthcare sector [21]. The Egyptian National Authority for Quality Assurance and Accreditation of Education emphasized the importance of nurses in the advancement, preservation, and restoration of health. As a result, the Egyptian healthcare sector determined that training competent nurses with advanced nursing competencies is essential. For nurses to fulfill their essential responsibilities as health promoters, educators, counselors, care coordinators, case managers, and researchers, they must possess these competencies [22].

Evaluating nurses’ clinical competence to identify their educational needs and taking appropriate action to improve are the most crucial elements in raising nurses’ professional competence and, as a result, improving the standard of nursing care [1, 17]. Furthermore, nurses must learn how to evaluate their knowledge and knowledge gaps to maintain their competence and safety in the workplace [23]. Additionally, through critical thinking, self-assessment empowers nurses to participate more actively in the learning process and promotes continuous learning [24, 25].

Various studies have examined nurses’ clinical competence worldwide. As far as researchers are aware, there is a notable lack of comprehensive published research specifically addressing the clinical competence of registered nurses within the healthcare context in Egypt. Most existing studies focus on inpatient care, leaving a gap in understanding how nurses perform in outpatient settings, particularly given the unique challenges and demands of outpatient care. This underscores the necessity of this study.

### Aim

This study aimed to measure the level of self-evaluated clinical competence and its relationship with demographic and occupational parameters among registered nurses employed at selected outpatient clinics in Egypt.

### Research questions

The following questions were the focus of this study.

1. What is the level of a nurse’s perceived clinical competence?
2. What is the relationship between the mean score of clinical competence and demographic and occupational factors?

## Materials and methods

### Ethics approval and consent to participate

The researchers conducted the study following the guidelines and ethical standards established by the Research Ethics Committee of the Faculty of Nursing at Mansoura University (IRB no: P.0382). This research adhered to the ethical guidelines of the 1964 Helsinki Declaration and any subsequent revisions. Permission for data collection was granted by the hospital’s nursing administrations. Participants were informed about the study’s purpose and assured of the confidentiality of their data and informed written consent was obtained. The first page of the questionnaire indicated that participants had given written approval after being informed. The study was a fully voluntary, non-profit endeavor, and participants were informed of their right to withdraw from the study at any time without explanation. Participants also received assurances regarding the privacy of their responses and requirements. Each participant’s data was input into an electronic database using a secure computer and internet connection, which was password-protected. The electronic database was accessible only to specific members of the research team. Regular automated backups were performed to monitor data entry. Throughout the study, all participant records were securely maintained by the chief investigator. Code numbers were created and managed by the researchers.

### Study design and setting

A descriptive, cross-sectional study was conducted to determine the prevailing characteristics in a population at a certain point in time, making it the best way to determine prevalence and study associations of multiple exposures and outcomes [26]. To ensure high-quality reporting, the study followed the requirements of the Strengthening Reporting of Observational Studies in Epidemiology (STROBE) [27] (S1 Checklist in Supporting Information).

The study took place at the outpatient clinics of two governmental hospitals (Mansoura University Hospital and Mansoura Chest Disease Hospital) and five primary healthcare centers (MCH Talkha, Meet Khamis, Awal Almansoura, Meet Mazah, and Salamoon Elkomash) in Mansoura city, Egypt between January 18 and June 22, 2023. These hospitals cater to both adult and pediatric patients, offering a wide range of services, while primary healthcare centers provide comprehensive care for all ages, including palliative care, promotion, treatment, rehabilitation, and prevention.

### Participants

The participants in this study were chosen based on the following specific inclusion criteria: (1) registered nurses employed in various clinical settings within any associated Ministry of Health and Population (MOHP) healthcare institution for a minimum of 12 months as they transitioned to become competent nurses; (2) they were aged between 23 to <60 years, regardless of sex; (3) had at least a baccalaureate degree in nursing sciences; and (4) willingly consented to participate in the study. Still, over 60 nurses were working in administrative roles (managers, supervisors, etc.), nurses who had worked for fewer than six months, those who declined to participate in the study in writing, and those who indicated that they would not be answering the questionnaire were not included in the current study. The study included all participants who returned the questionnaire with at least 70% of the items completed. The sample size was calculated using Cochran’s formula. The study, which involved 230 nurses in Iran, yielded the mean score and standard deviation (2.82±0.53) of nurses’ clinical competence. Additionally, a 95% level of confidence and a 15% nonresponse rate were assumed. The study’s computed sample size, based on the given assumptions, was 450. Nonetheless, all the nurses who worked in the corresponding wards at each hospital and PHC were involved in boosting the study’s power and accuracy. The study used a census approach to include study participants. Participants were selected using the purposive sample technique to ensure that the results represented the population. According to Polit and Beck [28], purposive samples have attributes based on a certain feature that will help the study.

### Study variables

The clinical competency of the nurses was the dependent variable, while, the study’s independent factors included socio-demographic and occupational characteristics.

### Instruments

A structured, self-administered two-part Arabic questionnaire was used to collect data from the study participants. Each question required a closed response.

The first part gathered participants’ demographic and occupational information including their age, sex, highest level of nursing education (bachelor’s, diploma, or master’s), work location, and years of work experience.

The second part focused on the Clinical Competence Questionnaire (CCQ), which evaluates perceived nurses’ clinical competency in various clinical settings. The CCQ was developed by [29], who validated the content and known-group validity of the study. The author of the study reported a Cronbach’s alpha of 0.98 for the complete CCQ. We used a translated version of the questionnaire to compensate for the nurses’ limited command of the English language. To address the nurses’ limited English proficiency, we utilized a translated version of the questionnaire. Three independent translators were tasked with translating the original version of the questionnaire into Arabic. A condensed version was selected through consensus from the three Arabic translations. The final Arabic form underwent linguistic and structural review. The format and number of questions in the Arabic version of the questionnaire matched those of the original English version. No grammatical issues were found in the translation and cultural adaptation was unnecessary as the questionnaire items were culturally relevant. The test-retest reliability of the Arabic version in this study was 0.910.

The CCQ (46 items) consists of four main competency domains with corresponding and specific competencies required for nurses: (a) nursing professional behaviors, which include 16 competencies; (b) general performance, which includes 12 competencies; (c) core nursing skills, which include 12 competencies; and (d) advanced nursing skills, which include six competencies. A Likert scale was used for scoring with five domains ranging from one (for incapacity to perform) to five (for theoretical concept fluency and practical performance under supervision). A higher score indicates a person’s self-perception of a better level of clinical competence. The total score ranges from 46 to 230. Participants’ overall clinical competence scores were categorized as competent enough if they fell between 80 and 100% (184–230), somewhat competent if they fell between 60–79% (138–181.7), and slightly competent if they were below 60% (138), using a modified Bloom’s criteria cutoff point [30].

Before collecting the data, the nursing faculty at Mansoura University and selected healthcare facilities obtained official approval. Two researchers visited and contacted the nursing departments of each hospital and healthcare facility to arrange suitable times and dates for data collection. After meeting with the nursing department, the researcher scheduled meetings with all unit managers to explain the study and obtain consent to distribute the questionnaire to participants. A list of nurses’ names was then obtained by a researcher from the nursing office.

The aim of the study was then communicated to qualified nurses based on their shift patterns. Nurses were asked to participate in the study if they felt satisfied with the researchers’ explanations. After receiving written informed consent, the nurses were given the questionnaire by the researchers, which was then collected. It took the nurses fifteen to twenty minutes to complete the questionnaire.

Ten percent of the study sample (n = 45), who were not initially included in the study, took part in a pilot trial. The purpose of the pilot study was to assess the questionnaire’s usability, clarity, and feasibility as well as to determine the time required for completion. It also aimed to identify any barriers or issues that could hinder data collection. No modifications were made.

In this study, several measures were taken to enhance the quality of the data collection process: participants were provided with clear explanations of the study’s aim and had their questions answered.

Additionally, participants were given sufficient time to complete the questionnaire, it was administered at a convenient time, and surveys with more than 20% missing data were excluded. The response rate for the questionnaire was 100%.

The data-gathering technique was pretested, and the researchers received comprehensive training, to ensure uniformity and standardization of data collection processes. The dependability of the tool was confirmed by checking its content, face, and construct validity, as well as ensuring that the tool’s interclass correlation coefficients (ICC) were greater than 0.7.

### Data analysis

Version 25 of the Statistical Package for Social Sciences (SPSS) (Armonk, NY, USA) [31] was used to analyze the data after visualization, organization, removal of the outliers from the data set, and tabulation of the collected data in S1 Raw data. The normality of the data was checked using the Shapiro-Wilk and Kolmogorov-Smirnov tests [32]. The variables were examined for anomalies, missing data, and possible errors. Every statistical analysis involved the verification of assumptions. The study utilized both descriptive and inferential statistics, specifically the independent sample t-test, mean, standard deviation, frequency, percentage, and chi-square statistics. An independent sample t-test was used to compare the mean clinical competence in terms of dual-mode qualitative variables. To control for potential confounding variables, an Analysis of Covariance (ANCOVA) was employed. The Cronbach’s alpha coefficient test was utilized to determine the reliability (internal consistency) of the questionnaires used in the study. An acceptable level of internal consistency was defined as having a Cronbach’s alpha between 0.6 and 0.7, good internal consistency as 0.7–0.9, and outstanding internal consistency as >0.90 [33, 34]. Statistical significance was considered to have occurred at a P-value ≤ 0.05.

## Results

### Participant characteristics

The study sample consisted of 450 nurses with a mean age of 28.7± 7.37 years, with more than three-quarters of them 76.7% being females. Additionally, 86.7% worked in government hospitals, and 80% held a bachelor’s degree in nursing sciences. Furthermore, less than two-thirds (61.3%) had more than 10 years of clinical experience, as shown in Table 1.

**Table 1:** Distribution of the participants’ demographic and occupational data.

Demographic and Occupational Data	N = 450	%
**Age (years) $\bar{x}\pm S.D$**	28.7± 7.37	
<25 years	159	35.3
25<35 years	216	48
35<55 years	75	16.7
**Sex**		
Female	345	76.7
Male	105	23.3
**Level of nursing education**		
Bachelor’s degree	360	80
Diploma degree	27	6
Post-graduate degree	63	14
**Work setting**		
Governmental hospital	390	86.7
Primary healthcare center	60	13.3
**Years of clinical experience**		
<5 years	54	12
5–10 years	120	26.7
>10 years	276	61.3

Table 2 shows the distribution of participants based on their level of clinical competence. In terms of the domain of professional behaviors, the study found that 54.9% and 83.3% of the participants were competent enough to follow health and safety precautions and act to prevent or reduce the risk of infection for patients, respectively. However, only 2.2% of participants demonstrated competence in accepting cultural diversity. On the other hand, 78.7% of participants were competent enough in applying critical thinking to patient care.

**Table 2:** Distribution of the participants according to their levels of clinical competence.

Domains of Nursing Competence	Levels of Clinical Competence			
	Very competent	Competent enough	Somewhat competent	Slightly competent
**I. Professional Behaviors**				
Follow health and safety precautions	162(36)	247(54.9)	35(7.8)	6(1.3)
Take appropriate measures to prevent or reduce the risk of self-harm	3(0.7)	342(76)	78(17.3)	27(6)
Take appropriate measures to prevent or reduce the risk of infection for patients	63(14)	375(83.3)	3(0.7)	9(2)
Implement appropriate measures to prevent problems affecting patient	132(29.3)	264(58.7)	45(10)	9(2)
Abide by instructions for maintaining the confidentiality of information of patients	3(0.7)	321(71.3)	54(12)	72(16)
The application of accepting the diversity of cultures	120(26.7)	309(68.7)	11(2.4)	10(2.2)
Adhere to ethical and legal standards of nursing practice	120(26.7)	159(35.3)	129(28.7)	42(9.3)
Maintain appropriate appearance, dress, and behavior	10(2.2)	362(80.4)	68(15.1)	10(2.2)
Understand the rights of the patient	114(25.3)	147(32.7)	72(16)	117(26)
Recognize and maximize learning opportunities	81(18)	291(64.7)	75(16.7)	3(0.7)
Apply appropriate measures and resources to solve problems	183(40.7)	192(42.7)	51(11.3)	24(5.3)
Apply or accept constructive criticism	66(14.7)	330(73.3)	51(11.3)	3(0.7)
Apply critical thinking to patient care	48(10.7)	354(78.7)	45(10)	3(0.7)
Oral communication in an accurate and promptly with patients	54(12)	267(59.3)	111(24.7)	18(4)
Oral communication in accurate and timely terms with healthcare professionals	129(28.7)	291(64.7)	9(2)	21(4.7)
Understand and support the objectives of the working group	153(34)	180(40)	75(16.7)	42(9.3)
**II. General Performance**				
Taking the health history of the new patient	81(18)	258(57.3)	12(2.7)	99(22)
Conducting and documenting the patient’s health assessment	102(22.7)	255(56.7)	9(2)	84(18.7)
Answer questions from patients or their families	153(34)	183(40.7)	105(23.3)	9(2)
Educate patients or families with knowledge about disease care	117(26)	234(52)	21(4.7)	78(17.3)
Graphics work and documentation	63(14)	327(72.7)	6(1.3)	54(12)
Develop a patient care plan	90(20)	279(62)	6(1.3)	75(16.7)
Shift report performance	126(28)	219(48.7)	99(22)	6(1.3)
Hand washing and daily routine care	40(8.9)	396(88)	8(1.8)	6(1.3)
Provide comfort measures	75(16.7)	306(68)	63(14)	6(1.3)
Nutrition and fluid balance assessment	3(0.7)	300(66.7)	132(29.3)	15(3.3)
Evaluate the output status	6(1.3)	351(78)	90(20)	3(0.7)
Assisting the patient with activities and mobility, and changing position	3(0.7)	348(77.3)	93(20.7)	6(1.3)
**III. Core-Nursing Skills**				
Provide emotional and psychological support	3(0.7)	315(70)	129(28.7)	3(0.7)
Intravenous puncture with canola	90(20)	300(66.7)	6(1.3)	54(12)
Intravenous administration	117(26)	267(59.3)	21(4.7)	45(10)
Change the bottle or bag of intravenous fluids	81(18)	357(79.3)	6(1.3)	6(1.3)
Giving medicines intravenously (or in IV bags)	81(18)	327(72.7)	30(6.7)	12(2.7)
Intramuscular administration of drugs	170(37.8)	260(57.8)	10(2.2)	7(0)
Subcutaneous (or intradermal) injection	138(30.7)	306(68)	3(0.7)	3(0.7)
Giving medicines orally	96(21.3)	340(75.6)	3(0.7)	11(2.4)
Blood transfusion	114(25.3)	300(66.7)	27(6)	9(2)
Urinary catheter insertion and care procedure	102(22.7)	330(73.3)	6(0.6)	12(2.7)
Perform sterilization techniques and measures	81(18)	129(28.7)	96(21.3)	144(32)
Postural drainage and oxygen therapy procedures	9(2)	231(51.3)	180(40)	30(6.7)
**IV. Advanced Nursing Skills**				
Perform preoperative/postoperative care	54(12)	162(36)	231(51.3)	3(0.7)
Upper airway suction performance	11(2.4)	109(26.7)	305(73.3)	25(50)
Laryngeal tube care procedure	10(2.2)	110(24.4)	315(70)	15(3.3)
Nasogastric tube feeding and care procedure	13(2.9)	80(17.7)	360(80)	7(1.5)
Chest tube care procedure	12(2.7)	60(13.3)	370(82.2)	8(1.8)
Caring for a wound dressing	54(12)	123(73.3)	189(42)	84(18.7)

In the domain of general performance, over half of the participants, 52% were competent enough to educate patients or families on disease care. Additionally, 8.9% of them were highly competent in handwashing and daily routine care. However, only 3.3% were slightly competent in conducting nutrition and fluid balance assessments.

In terms of the participants’ competence in core nursing skills, it was discovered that 70% were competent enough to provide emotional and psychological support to patients, while 66.7% were skilled enough to administer blood transfusions. However, only 32% showed slight competence in carrying out sterilization techniques and procedures.

When it comes to advanced nursing skills, the study found that 51.3% of participants were somewhat competent in providing preoperative and postoperative care. Additionally, 73.3% of them were deemed competent in wound dressing care. However, only 1.8% of participants were considered slightly competent in performing chest tube care procedures.

**Fig 1:**
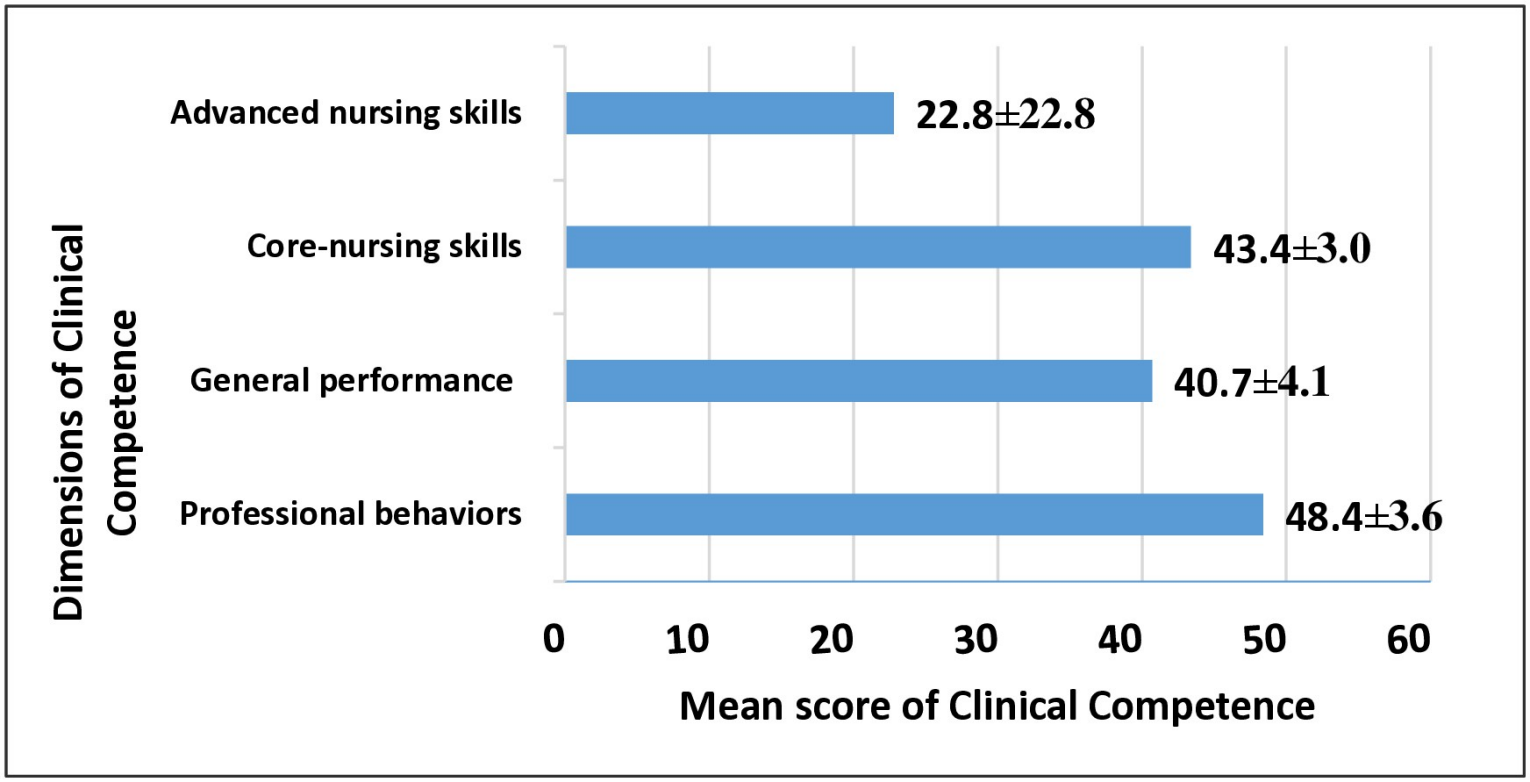
Distribution of the participants according to their mean score of clinical competence domains.

The average score for clinical competence among the participants is presented in Fig 1. In terms of professional behaviors and general performance, the average score for clinical competence was 48.4±3.6 and 40.7±4.1 respectively. Additionally, the average score for clinical competence regarding core and advanced nursing skills was 43.4±3.0 and 22.8±1.5 respectively. Overall, it was found that the nurses displayed a moderate level of clinical competence (155.3±7.2 out of 230).

The relationship between the mean clinical competence score of participants and their occupational and demographic variables is presented in Table 3. The mean clinical competence score of nurses was found to be highly significant (*P*<0.001) concerning their age, with the nursing professional behaviors domain scoring the highest. Furthermore, a significant correlation was observed between the nurses’ level of nursing education and their mean clinical competence score (*P*<0.001), with the nursing professional behaviors category scoring the highest. It was also evident that the average clinical competency score was strongly correlated with the number of years of clinical work experience the nurses had (*P*<0.001), with the nursing professional behaviors domain receiving the highest score.

**Table 3:** Relation between the participants’ demographic and occupational characteristics and their mean score of clinical competence domains.

Nurses’ demographic characteristics	Nursing Professional Behaviors	General Performance	Core Nursing Skills	Advanced Nursing Skills
**Age (years)**				
< 25	28.5±4.3	20.7±3.1	20.4±3.8	6.3±2.9
25<35	33.9±7.9	24.2±5.9	23.1±5.1	7.6±3.1
35<55	36.9±8.8	24.4±6.4	26.8±8.1	8.9±2.9
**Signif. test**	F = 51.6, *P*<0.001*	F = 33.2, *P*<0.001*	F = 27.4, *P*<0.000	F = 28.4, *P* = 0.00*
**Sex**				
Female	31.9±7.2	22.7±5.2	22.5±5.1	7.2±3.2
Male	34.3±9.1	24.1±6.1	23.4±7.3	8±3.1
**Signif. test**	t = 7.3, *P* = 0.007*	t = 5.32, *P* = 0.023*	t = 1.9, *P* = 0.166	t = 4.5, *P* = 0.034
**Work setting**				
Government hospital	32.6±7.8	23.3±5.6	22.3±5.7	7.3±3.3
Primary Healthcare	32±7.1	21.4±4.2	25.3±5.2	8.1±2.4
**Signif. test**	t = 0.33, *P* = 0.566	t = 6.3, *P* = 0.013*	t = 13.1, *P*<0.001*	t = 2.2, *P* = 0.131
**Level of nursing education**				
Bachelor (BSc)	31.7±7.6	23.2±5.2	21.9±5.1	7±3.1
Post-graduate degree	32.1±4.1	18.6±2.1	23.4±5.4	8.1±1.9
Diploma	43.6±6.3	31.1±3.6	31.6±7.4	10.4±3.7
**Signif. test**	F = 22.6, *P*<0.001*	F = 74.5, *P*<0.001*	F = 43.9, *P*<0.000	F = 21.2, *P*<0.001*
**Years of clinical work experience**				
<5 years	34.1±7.1	23.9±5.6	23.3±4.8	7.6±3.2
5–10 years	27.9±5.8	19.3±2.1	20.2±5.3	6.7±2.4
>10 years	35.1±9.6	26.7±5.7	25.6±8.5	7.7±4.2
**Signif. test**	F = 41.7, *P*<0.001*	F = 59.2, *P*<0.001*	F = 30.2, *P*<0.001*	F = 5.8, *P* = 0.005*

F: ANCOVA. *P*: Significance

* Significant (*P*<0.05)

** Highly significant (*P*<0.001)

## Discussion

Global healthcare systems aim to provide high-quality care at a reasonable cost with universal access for all populations [35]. Given the local nursing shortage, it is crucial to ensure that hired nurses are qualified to deliver quality nursing care, especially concerning patient safety and treatment [36]. Competency assessment is a critical issue for all involved in healthcare, including managers, educators, policymakers, and nursing professionals [37].

The current study’s findings revealed that nurses had a moderate overall level of clinical competence according to their average score, as perceived by nurses themselves. This moderate level of competence may be due to organizational issues, resource limitations in outpatient clinics, or training deficiencies. They demonstrated proficiency in the core competence areas of nursing skills, general performance, and professional behaviors as they form the backbone of nursing practice and are considered the set of ethical standards and codes of conduct that are expected to be adhered to in a professional setting but scored poorly in advanced nursing competence. Nurses may prioritize areas closest to direct patient care, leading to differences in competency scores.

When assessing their performance, individuals often rate themselves higher, and the nurses in this study displayed a strong sense of confidence in their abilities. Similar conclusions were drawn in studies from Jordan [38], China [39], and Ethiopia [40], where nurses believed their competence level to be average or slightly above average. Contrary to the findings of the current study, a study conducted in Italy by [41] revealed that the degree of clinical competencies and the extent to which nursing skills are employed by nurses were high. These outcomes contradict the research findings of studies conducted in Iran [42], and Europe [43], which indicated that nurses assigned a very high rating to their competence. Furthermore, a Swedish study conducted by [44] found that nurses’ assessments of their clinical competency were lowest in areas of professional development and direct clinical practice and highest in areas related to team collaboration and ethics. Additionally, a study by [45] revealed that nurses who rated their competence as the lowest had high workloads, unsatisfactory jobs, and experiences with both financial and professional insecurity.

The level of competency varies across different domains of competence. For example, in a study using a different scale, nurses rated their level of competency highest in organizing and implementing nursing care and in following ethical and legal standards of nursing practice [46]. The study found a strong correlation between nurses’ clinical competence and various occupational and demographic factors, such as age, gender, workplace setting, level of nursing education, and years of clinical experience. Nurses who are older, female, work in government hospitals, have a bachelor’s degree in nursing, and have more than ten years of clinical experience tend to have higher levels of clinical competence. One plausible explanation for this could be that nurses’ ages, educational backgrounds, and work experiences are all on the rise, which could lead to an improvement in their clinical competence. Due to curriculum-required competencies, nurses with a bachelor’s degree in nursing were anticipated to possess superior abilities and knowledge, and they are also more likely to have received additional training. The findings of the present study corroborated those of the studies conducted by [37, 47], which discovered a substantial correlation between age and clinical competence. However, the findings of this study are at odds with those of studies conducted in Ethiopia [48], and Iran [49], which reported that there was no statistically significant correlation between gender variable and mean clinical competence. Moreover, these findings contradict those of [50, 51], who found no connection between clinical competence and the sex variable.

Higher self-assessed competence among registered nurses in post-graduate programs has been linked to higher academic degrees [52]. However, this conflicts with the findings of the current study, which showed that nurses with bachelor’s degrees believed they had a higher clinical competence score. This result is understandable, as many of the nurses in the study held bachelor’s degrees in nursing. This result makes sense. Additionally, these results contradict those of a previous Iranian study [49], which found that nurses with MSc degrees had a higher mean clinical competence than nurses with BSc. Degrees, despite the lack of a statistically significant difference.

Previous Ethiopian studies have shown that a nurse’s competency varies according to the duration and frequency of their work experience [40, 48]. The current study discovered a statistically significant relationship between years of clinical work experience and the degree of clinical competence. In actuality, the nurses’ work experience increases with their employment history. Therefore, it is anticipated that as work experience increases, clinical competence will also rise. This may be because they have had more exposure to different patients and situations, and they have also had the opportunity to learn from their mistakes. A similar conclusion was reached by another study [41]. On the other hand, the study by [53] disagrees with the findings of this study because it suggests that factors such as a high workload, job satisfaction, and salary contribute to nurses’ impairment in their line of work. As a result, they are not satisfied with their jobs, and this situation worsens as the age and work experience thresholds increase. However, a systematic review conducted by [54] found that environmental-organizational factors, identification of patients’ culture, provision of care based on their culture, job satisfaction, and consultation with colleagues were effective in improving nurses’ clinical competencies. Additionally, in nursing practice, developing trust, using adaptive care techniques, and fostering interdisciplinary teamwork are essential. Healthcare organizations should provide conditions that help nurses advance their careers and deliver effective, person-centered care [54].

We acknowledge that cultural issues significantly influence nurses’ judgments of clinical competence in the Egyptian healthcare system. The way Egyptian culture views hierarchy, deference to authority, and gender conventions may affect how nurses view themselves and are viewed by other members of healthcare teams. Furthermore, cultural perspectives on lifelong learning, career advancement, and the role of nursing in the healthcare system might influence an individual’s perception of their abilities as well as assessments from other sources.

## Conclusion

Based on the self-reported results of the nurses, our study found that their level of clinical competency was moderate based on average scores. There is a strong correlation between nurses’ clinical competency levels and occupational and demographic characteristics, such as age, sex, type of work environment, degree of nursing education, and years of clinical experience. These findings highlight the importance of implementing targeted training programs, potentially utilizing effective modalities like e-learning, role-playing, or simulations, known to enhance clinical competence. Policymakers, nurse educators, and healthcare providers can use this knowledge to promote nurses’ clinical competencies through intervention measures and foster a culture of continuous learning. Advanced nursing skills can be improved through knowledge enhancement, skill acquisition, ongoing training, emotional intelligence, and constructive feedback. Our findings support the notion that competence grows and improves with experience in real-world settings. To optimize nurses’ clinical competencies, future studies and interventions should focus on exploring the impact of specific training interventions, such as, role play, and problem-based learning, on nurses’ clinical competence, or conduct comparative studies across various healthcare settings.

## Recommendations

The study recommends that healthcare organizations create educational interventions to improve nurses’ clinical competence. These interventions should include organized and systematic feedback mechanisms, comprehensive training programs, mentorship programs, and continuous opportunities for professional development. By implementing these measures, nurses will be able to stay up-to-date on the latest procedures and technological advancements, cultivate a positive learning environment, and address the specific challenges faced by nurses of different genders. Moreover, to assist stakeholders such as healthcare industry managers, legislators, nurse educators, and nursing faculties in meeting the expectations and demands for competence, it is essential to engage in practicing advanced nursing skills and conducting ongoing evaluations of clinical competence. Effective management improvement and educational planning are necessary for nurses to enhance their clinical competence.

### Implication of the study

The significance of this research is immense because the nursing profession relies on nurses’ clinical competence to provide high-quality care and improve patient outcomes. The study’s results provide current information on the level of clinical competence among nurses and the factors that influence it. Policymakers, program designers, and other researchers interested in conducting interventions to improve nurses’ clinical competence will find this information valuable. Local healthcare institutions, scholars, nursing societies, and other stakeholders working to elevate the standard of nursing care can also benefit from the study’s findings and find measures to apply to the Egyptian nursing community.

## Strengths and limitations of the study

The study is the first of its kind in Egypt, aiming to measure the level of clinical competence and its relationship with demographic and occupational parameters among registered nurses. Since measurement instruments for assessing nursing clinical competence are limited, the research findings may be of great interest to the professional community.

Despite this valuable contribution, the study has several limitations that need to be considered when interpreting the findings. Firstly, the non-probability purposive sample used in the study raises the possibility of bias. Secondly, there is a potential selection bias as those who did not participate in the questionnaire may have different responses compared to those who did. Thirdly, assessing clinical competence based on self-reported responses from participants may lead to over-reporting or information bias, which reduces confidence in the findings. Additionally, the participants’ employment situations were not sufficiently large or evenly dispersed to allow for generalization of the results.

However, by identifying areas of strength and potential growth through self-assessment, nurses can enhance their practice. Therefore, it would have been beneficial to incorporate information obtained through objective methods alongside self-assessment. To address these limitations, the authors of this study attempted to minimize bias by having a non-biased scorer consistently score the questionnaire. Future research should consider other study designs, such as longitudinal and intervention studies, to further explore this topic.

## Supporting information

S1 Checklist STROBE statement—Checklist of items that should be included in reports of cross-sectional studies. (PDF)

S1 Raw data (XLSX)
